# Recent Progress in Chemo-Enzymatic Methods for the Synthesis of N-Glycans

**DOI:** 10.3389/fchem.2020.00513

**Published:** 2020-06-16

**Authors:** Qiang Chao, Yi Ding, Zheng-Hui Chen, Meng-Hai Xiang, Ning Wang, Xiao-Dong Gao

**Affiliations:** Key Laboratory of Carbohydrate Chemistry and Biotechnology, Ministry of Education, School of Biotechnology, Jiangnan University, Wuxi, China

**Keywords:** N-glycosylation, chemo-enzymatic synthesis, glycosyltransferases, glycosidase, glycosynthase, homogeneous glycoprotein, biomarker

## Abstract

Asparagine (N)-linked glycosylation is one of the most common co- and post-translational modifications of both intra- and extracellularly distributing proteins, which directly affects their biological functions, such as protein folding, stability and intercellular traffic. Production of the structural well-defined homogeneous N-glycans contributes to comprehensive investigation of their biological roles and molecular basis. Among the various methods, chemo-enzymatic approach serves as an alternative to chemical synthesis, providing high stereoselectivity and economic efficiency. This review summarizes some recent advances in the chemo-enzymatic methods for the production of N-glycans, including the preparation of substrates and sugar donors, and the progress in the glycosyltransferases characterization which leads to the diversity of N-glycan synthesis. We discuss the bottle-neck and new opportunities in exploiting the chemo-enzymatic synthesis of N-glycans based on our research experiences. In addition, downstream applications of the constructed N-glycans, such as automation devices and homogeneous glycoproteins synthesis are also described.

## Introduction

In living cells, oligosaccharides usually attach to other macromolecules, forming glycoconjugates to fulfill their biological functions (Hanson et al., 2004). As the representative glycoconjugate, glycoproteins are believed to constitute 50% of human proteins, which is still considered as an underestimated value (An et al., 2009). N-Linked glycosylation is one of the most abundant and complicated posttranslational modifications of proteins, which affects various biological processes such as lectin (calnexin/calreticulin)-mediated protein folding in the endoplasmic reticulum (ER) quality control system and the ER-associated degradation pathways (Helenius and Aebi, 2001; Roth and Zuber, 2017). N-Glycans also play important roles in signal transduction, embryogenesis, neural development, immune regulation, and cell proliferation (Moremen et al., 2012), and they are associated with pathogen recognition, immune responses, autoimmune diseases, cancer cell proliferation, and metastasis in pathological conditions (Ohtsubo and Marth, 2006; Lauc et al., 2016). Therefore, obtaining diverse glycan structures is essential to study their biological roles and can be further applicable in the glycoprotein synthesis (Pilobello and Mahal, 2007; Wang and Lomino, 2012; Hofmann and Pagel, 2017; Hyun et al., 2017).

The structures of oligosaccharides are far more diverse and complex than those of nucleic acids and proteins, due to the variety of monosaccharide residues and linkages of the glycosidic bonds (Bertozzi and Rabuka, 2009). The assembly and digestion of N-glycans are catalyzed by a series of glycosyltransferases (GTs) and glycosidases (GHs). In mammalian cells, more than 30 enzymes are involved in the trimming of N-glycans in the Golgi apparatus (Stanley et al., 2009), each of which may influence the glycan structures, leading to the heterogeneity of N-glycans. For example, the mouse zona pellucida glycoprotein bears up to 58 N-glycan structures at one N-glycosylation site (Stanley et al., 2009). In other words, naturally occurring glycans are always the mixture of extremely similar structures that are difficult to separate, making the preparation of the structurally well-defined oligosaccharides from natural sources impractical. This inadequacy led to a lack of in-depth studies on the molecular basis of how glycans regulate biological and disease processes (Hart and Copeland, 2010; Kiessling and Splain, 2010; Cummings and Pierce, 2014).

In recent decades, various synthetic methods including one-pot synthesis, solid-phase synthesis, cascade multienzymatic synthesis and chemo-enzymatic synthesis, have been well-investigated to prepare structurally defined N-glycans (Bartolozzi and Seeberger, 2001; Yu et al., 2005a; Muthana et al., 2009; Kajiwara, 2010; Bouhall and Sucheck, 2014; Yu and Chen, 2016; Kinnaert et al., 2017). However, it is difficult to achieve a general synthetic method for N-glycans due to their complicated structures and inherent chemical properties (Boltje et al., 2009). For instance, chemical synthesis which requires careful design in synthetic route and protecting groups, serves as the most reliable method to prepare structural well-defined oligosaccharides. Nevertheless, it is very time-consuming and sometimes risky to accomplish, especially for the highly complicated N-glycan structures. Enzymatic glycosylation is another efficient way to synthesize N-glycans, which doesn't require the introduction of protecting groups and can react under mild conditions with high specificity. But this method is still challenging because the suitable substrates are not always available. The development of chemo-enzymatic synthesis, which requires synthesized precursors and a series of corresponding GTs, has provided a novel approach to producing diverse N-glycans. Compared with chemical and enzymatic approaches, chemo-enzymatic methods show both high stereoselectivity and economic efficiency (Palcic, 2011; Schmaltz et al., 2011). In addition, automated solid-phase synthesis strategy, in which the enzymatic or chemical reactions occur automatically on a solid-phase carrier such as beads is expected to be broadly applicable thanks to its simplified purification steps. This review summarizes some recent advances in the chemo-enzymatic synthesis of N-glycans and discusses the applications and new opportunities in exploiting this method, which should be essential to understanding the roles of N-glycans in glycobiology.

## Enzymes and Donors Used in the Synthesis of N-glycans

**Figure 1:**
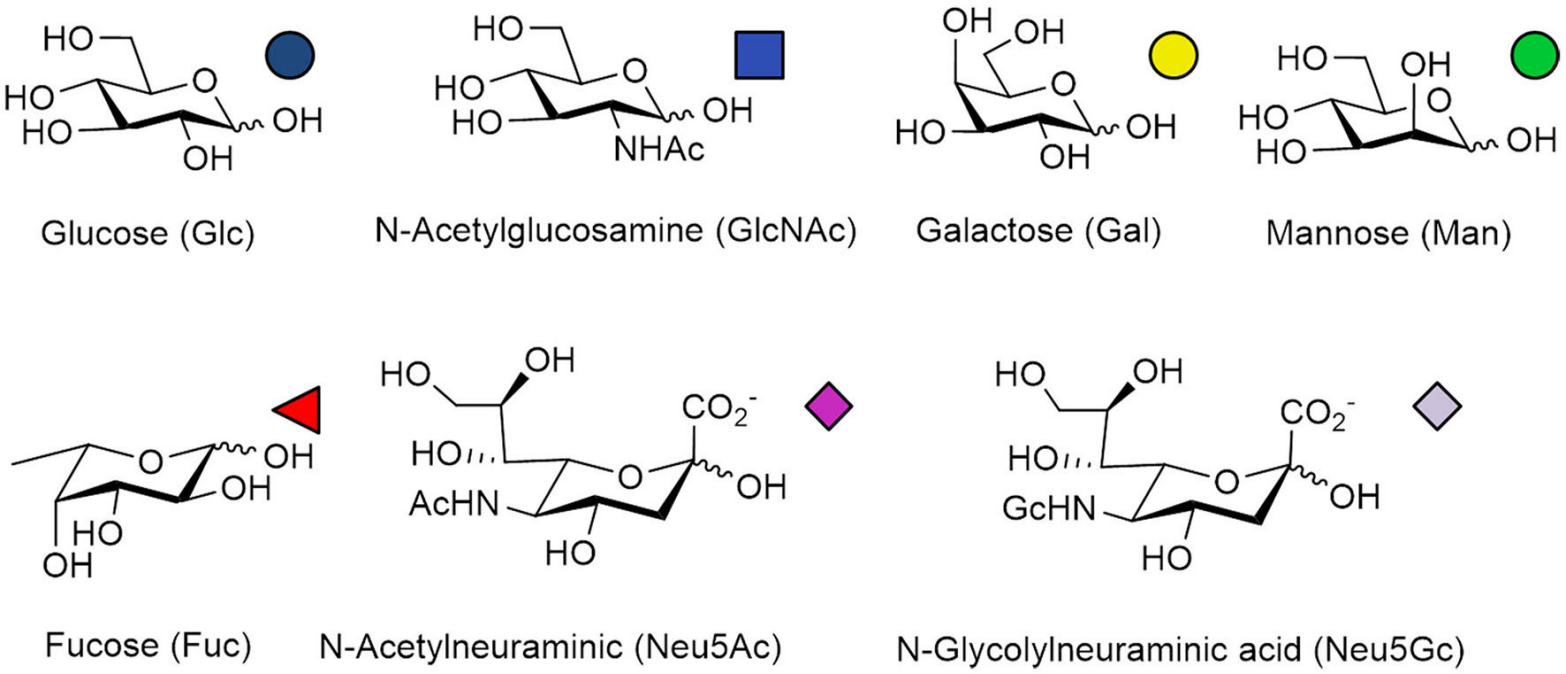
Structures and symbols of common monosaccharides in N-glycans.

N-Glycans widely exist in eukaryotic cells, whose common monosaccharide building blocks include N-acetylglucosamine (GlcNAc), mannose (Man), glucose (Glc), galactose (Gal), fucose (Fuc), and sialic acid (Neu5Ac, Neu5Gc). The structures and universal symbols of these monosaccharides are shown in Figure 1. There are also some unique monosaccharides in the N-glycosylation pathway in bacteria, archaea, fungi and plants (Deshpande et al., 2008; Nothaft and Szymanski, 2010; Eichler, 2013; Jarrell et al., 2014; Strasser, 2016). For example, N-acetylgalactosamine (GalNAc), glucuronic acid (GlcA), di-N-acetyl-d-bacillosamine (BacNAc_2_) and heptose (Hep) can be found in bacteria and archaea (Nothaft and Szymanski, 2010; Eichler, 2013; Jarrell et al., 2014). In plants, some N-glycans can be modified with xylose (Xyl) during their maturation process in the Golgi (Strasser, 2016). The highly specialized galactofuranose residues could be found in the N-glycan structures in the filamentous fungi (Deshpande et al., 2008). The N-glycans with these unique monosaccharide residues will not be discussed in this paper.

**Figure 2:**
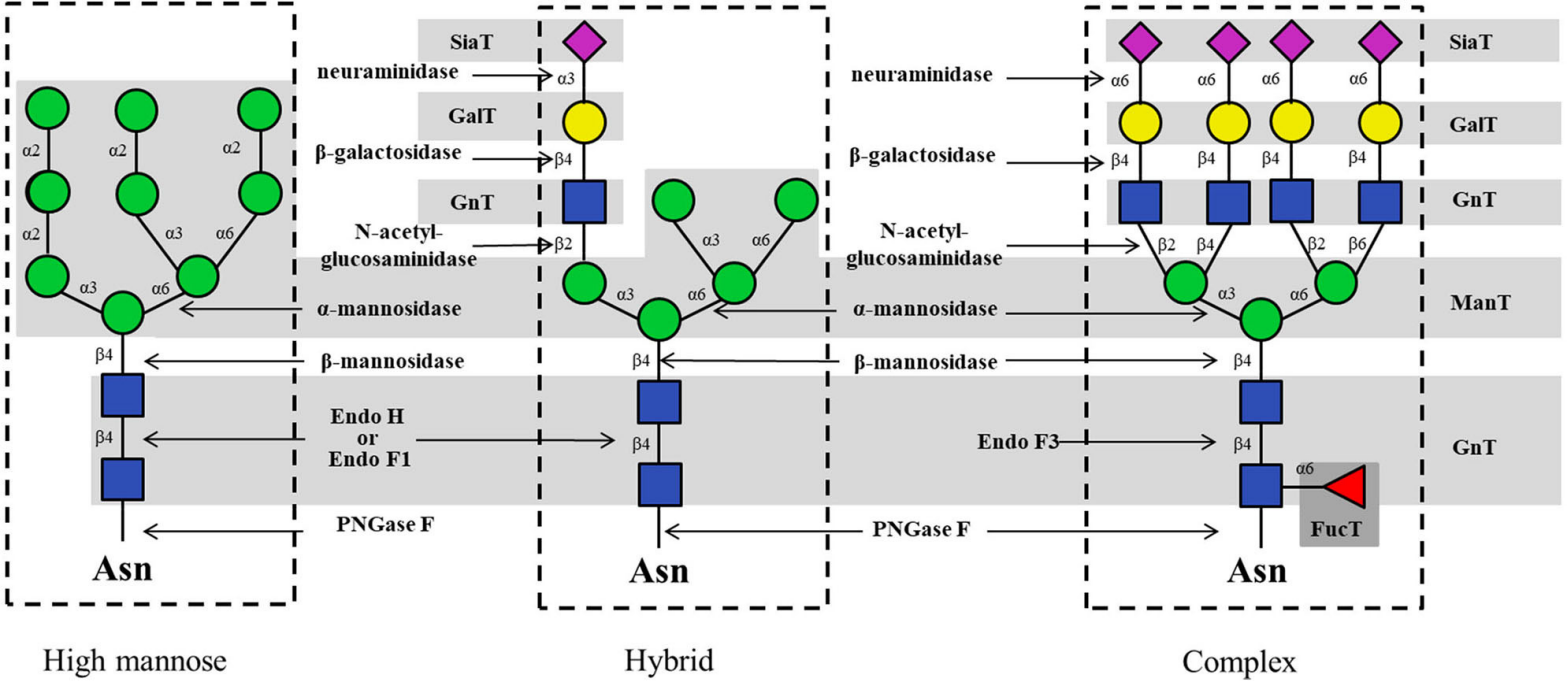
Typical glycosyltransferases and glycosidases for the enzymatic synthesis of high-mannose, hybrid and complex type N-glycans.

It is well-known that eukaryotic N-glycans can be structurally divided into high-mannose, hybrid and complex types (Figure 2). In the biosynthesis pathway, each type of N-glycan is assembled and trimmed stepwise by enzyme-catalyzed reactions involving GTs and GHs (Hanson et al., 2004; Schmaltz et al., 2011; Yu and Chen, 2016). Recently, the available number of these glycan-modifying enzymes is growing rapidly. Nearly 660000 GTs (classified into 110 families) and 770000 GHs (classified into 167 families) from all kingdoms of life can be found at the Carbohydrate-Active Enzymes (CAZy) database (http://www.cazy.org), indicating the potential to use some of these enzymes for the chemo-enzymatic synthesis of N-glycans. In contrast, the donors for N-glycosylation including sugar nucleotides and dolichol phosphate sugar, are sometimes difficult to prepare or commercially expensive, resulting in another critical concern in obtaining N-glycans.

### N-Glycosylation Enzymes

#### Glycosyltransferases (GTs)

GTs are the enzymes which promote glycosidic bond formation by catalyzing the transfer reaction of a saccharide from its activated form (likely a nucleotide sugar) to the acceptor. The acceptor should contain a nucleophile, such as certain carbohydrate hydroxyl groups or the nucleophile in a macromolecule (Breton et al., 2006; Lairson et al., 2008; Wagner and Pesnot, 2010). In recent decades, the number of characterized GTs, which allows a wide range of glycoside linkages to be installed for the preparation of specific N-glycan epitopes, has been increasing (Muthana et al., 2009; Schmaltz et al., 2011; Moremen et al., 2018).

Currently, researchers prepare recombinant GTs either in prokaryotic or eukaryotic expression systems. Taking the advantage of the high expression level, many GTs have been prepared in prokaryotic expression systems such as *Escherichia coli* (*E. coli*) as either soluble forms which may be full-length and truncated catalytic domains (Seto et al., 1995; Rao et al., 2009), or *in vitro* refolded precipitates (Ramakrishnan and Qasba, 2001). However, the prokaryotic expression system often produces nonfunctional protein aggregates (Paulson and Colley, 1989) when preparing eukaryotic proteins due to the lack of the protein modification system and chaperones for proper folding. Therefore, many GTs are expressed in eukaryotic expression systems, including mammals, insects and yeast (Ramirez et al., 2017; Moremen et al., 2018; Gao et al., 2019). It is worth mentioning that, recently, the eukaryotic expression constructs of all human glycosylation enzymes were generated (Moremen et al., 2018). In this strategy, a modular approach was used to create the library of the expression vectors, which were then transformed into mammalian or insect host cells for the protein expression. By removal of the transmembrane domains at the N-terminus or C-terminus, the active form of recombinant human GTs can be prepared with a high expression level. This work greatly expands the use of GTs for the synthesis of N-glycans (Prudden et al., 2017). Based on the above works, the number of commercially available GTs is increasing, which allows researchers to use the enzymatic method as an alternative way to modify glycans.

**Figure 3:**
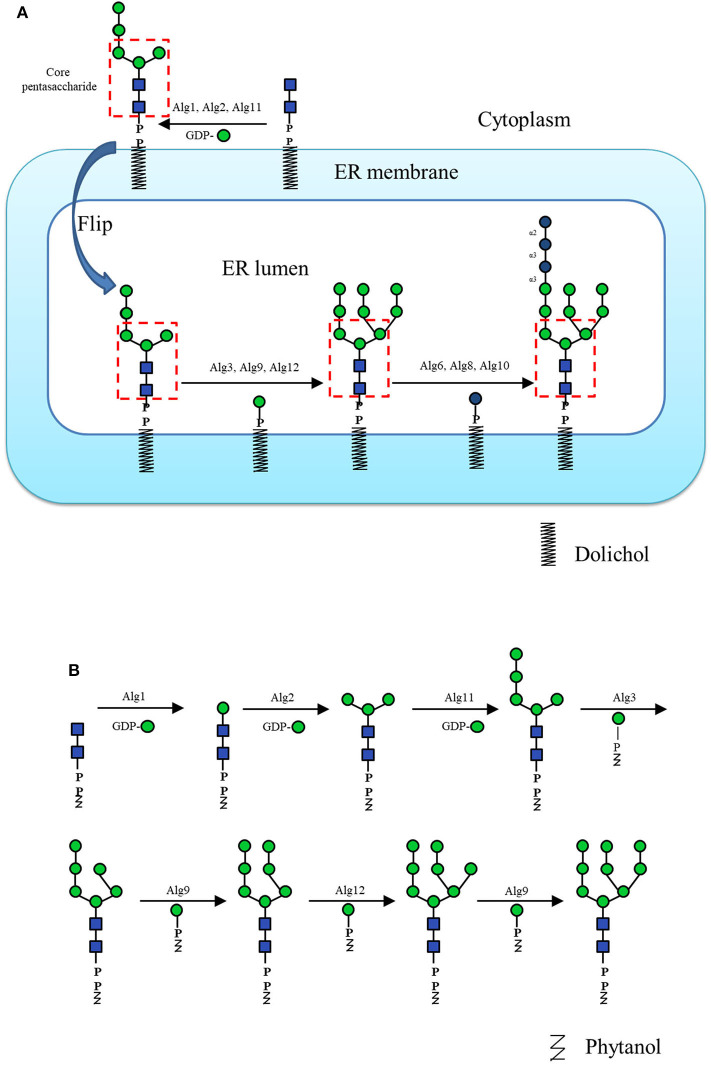
**(A)** The dolichol-linked oligosaccharide (DLO) biosynthesis pathway. **(B)** The schematic diagram of Man_9_GlcNAc_2_-PP-Phy enzymatic synthesis pathway *in vitro*.

In N-glycan enzymatic synthesis, 6 classes of GTs are commonly used, namely, N-acetylglucosaminyltransferases (GlcNAcTs), mannosyltransferases (ManTs), glucosyltransferases (GlcTs), galactosyltransferases (GalTs), fucosyltransferases (FucTs) and sialyltransferases (SiaTs) (Figure 2). In cells, assembly of the N-glycans is initiated by the biosynthesis of dolichol-linked oligosaccharide (DLO) in the ER. Before the transfer of oligosaccharides to nascent polypeptides, the glycan chain in the DLO is elongated sequentially by GlcNAcTs, ManTs and GlcTs (namely, Alg proteins), up to Glc_3_Man_9_GlcNAc_2_-PP-Dolichol, which contains 14 monosaccharide residues (Figure 3A). *In vitro*, since Flitsch and coworkers synthesized Man_1_GlcNAc_2_ by using recombinant β-1,4-ManT Alg1 in 1997 (Watt et al., 1997), the preparation of a series of DLO precursors such as the core pentasaccharide using corresponding Mans has been reported (Ramirez et al., 2017; Boilevin and Reymond, 2018).

In the biosynthesis pathway, the diversity of N-glycan structures starts to appear in the Golgi apparatus, where several GTs (i.e., GlcNAcTs, GalTs, SiaTs and FucTs) are involved in the N-glycan processing. GlcNAcTs, also called GnTs, increase the complexity of oligosaccharide structures by the modification of the core pentasaccharide Man_3_GlcNAc_2_ (Kizuka and Taniguchi, 2016), resulting in N-glycans with different numbers and linkages of branching GlcNAc moieties. These GlcNAc moieties could be converted to N-acetyllactosamines (LacNAc) by GalTs, leading to further structural diversity (Cummings, 2009). In the laboratory, the commercially available or expressed recombinant GnTs (e.g., GnT I, GnT II, and GnT IV) could be applied to the preparation of hybrid- and complex type N-glycan libraries including asymmetric multi-antennary complex type N-glycans by structurally remodeling microbial oligosaccharides, which could be used to generate the N-glycan microarrays for high-throughput screening of glycan-binding proteins (Hamilton et al., 2017).

In many glycoconjugates in living cells, sialic acid serves as the terminal epitopes of glycans. The formation of a certain sialyl glycosidic bond is quite difficult for *in vitro* chemical oligosaccharide synthesis due to the unique structure and properties of sialic acid (Schwardt et al., 2006). Therefore, SiaTs that transfer Neu5Ac groups from the donor CMP-Neu5Ac are particularly important for the chemo-enzymatic synthesis of N-glycans and glycoproteins. In 2017, Boons and coworkers prepared mono- and disialylated N-glycan derivatives using ST3-Gal-IV, a mammalian α-2,3-sialyltransferase, which recognized the LacNAc antenna structure as the sole substrate (Gagarinov et al., 2017).

In addition, fucose is also often found at the glycan terminus of many naturally existing glycoconjugates, such as the ABO and Lewis blood group epitope glycans. These fucoses are transferred from GDP-Fuc to the core or branch termini of N-glycans by FucTs, which are a series of unique GTs with high structural tolerance to donors and acceptors (Bastida et al., 2001; Khaled et al., 2004, 2008; Nguyen et al., 2007; Li et al., 2008; Woodward et al., 2010). This property enhances the utility and flexibility of FucTs in N-glycan synthesis *in vitro*. For example, after human FUT8 (an α-1,6 FucT) was overexpressed in a baculovirus system and purified, 77 structurally defined N-glycans were applied to the substrate specificity assay, which accordingly facilitated the development of an efficient chemo-enzymatic strategy to synthesize core-fucosylated asymmetric N-glycans (Calderon et al., 2016).

#### Glycosidases (GHs) and Glycosynthases

During its maturation process *in vivo*, the N-glycan is modified by first removing certain sugar residues (trimming), followed by re-glycosylation by appropriate GTs to give its mature form. Glycosidases, also called glycoside hydrolases, are enzymes that hydrolyze glycosidic bonds in glycans to saccharide units (Bourne and Henrissat, 2001). GHs are classified into endo-glycosidase and exo-glycosidase according to their cleavage sites in the oligosaccharide chain. Namely, an exo-glycosidase hydrolyzes the glycosidic bond at the non-reducing end of a glycan chain, whereas an endo-glycosidase hydrolyzes the internal glycosidic bond. Typical GHs for mammalian N-glycan trimming and their cleavage sites are summarized in Figure 2.

In the laboratory, trimming the N-glycans into desired structure is a valuable method in chemo-enzymatic glycan synthesis. Exo-glycosidases are efficient tools due to their capability to digest glycan structures. For instance, Fmoc labeled high-mannose type N-glycan Man_9_GlcNAc_2_Asn-Fmoc, which can be obtained from the Fmoc modified fractional precipitate of soybean flour, could be digested by the α-1,2-mannosidase to produce several high-mannose type N-glycan intermediates. Since Man_9_GlcNAc_2_-Asn-Fmoc contains four terminal Man-α-1,2-Man linkages, it can generate Man_5−8_GlcNAc_2_-Asn-Fmoc by the treatment with an α-1,2-mannosidase from the human gut bacterial symbiont *Bacteroides thetaiotaomicron* under controlled conditions (Toonstra et al., 2018). On the other hand, sialylated bi-antennary complex type N-glycan (SCT), which is available at large scale from sialoglycopeptide (SGP) isolated from the chicken egg yolk, can be further trimmed by sequentially adding sialidase, galactosidase, and N-acetylglucosaminidase to give various N-glycan structures. At present, several exo-glycosidases are routinely used to digest corresponding glycan bonds, most of which are commercially available or can be prepared in prokaryotic systems such as *E. coli* easily (Schmaltz et al., 2011).

**Figure 4:**
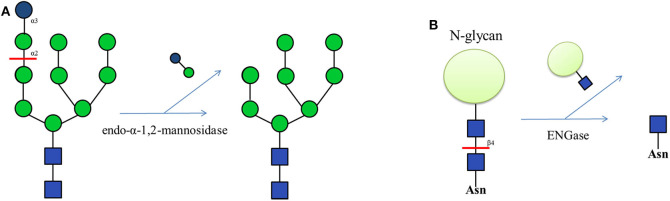
**(A)** The function of Golgi endo-α-1,2-mannosidase, which can cleave terminal Glc-Man moiety from GlcMan_9_GlcNAc_2_. **(B)** The function of endo-β-N-acetylglucosaminidase (ENGase), which can hydrolyze the N-glycan structure from glycoproteins.

Endo-glycosidases also show significant capacity in chemo-enzymatic methods to prepare N-glycans. Golgi endo-α-1,2-mannosidase, which can cleave the glucose-substituted mannose from immature glucosylated high-mannose type N-glycans (Figure 4A), is useful in chemo-enzymatic synthesis, such as the establishment of a high-mannose glycan library from a non-natural tetradecasaccharide precursor (Koizumi et al., 2013). Another widely used endo-glycosidase is endo-β-N-acetylglucosaminidase (ENGase), which hydrolyzes the N-glycan structure of the glycoprotein and leaves a single proximal GlcNAc residue (Figure 4B). ENGases from different species show substrate specificity toward N-glycan structures (Li and Wang, 2018). Endo D is specific for paucimannose (Man_1−3_GlcNAc_2_Asn); Endo A and Endo H specifically recognize the high-mannose type N-glycans; Endo F recognizes N-glycan structures ranging from the high-mannose to the bi-antennary complex type; and Endo M cleaves most N-glycan structures including the high-mannose, complex and hybrid types. Endos D, H and F are now commercially available (Schmaltz et al., 2011). Some ENGases show higher specificity, such as Endo S which cleaves only biantennary complex type N-glycan in the Fc domain of human IgG (Albert et al., 2008; Allhorn et al., 2010). In contrast, Endo S2 can cleave almost all kinds of N-linked glycans in IgG (Sjogren et al., 2013).

GHs are also suitable for the assembly of N-glycans since they sometimes possess the reverse ability of glycan transformation. For example, by using the substrate with a methylene linker between the glycan and peptide, Endo A could synthesize the high-mannose type C-linked glycopeptide with 26% yield (Wang et al., 1997). In another case, Endo M was employed to synthesize the glycopeptides containing high-mannose type N-glycans in 8.5% yield (Haneda et al., 1998). However, undesired side reactions, such as self-condensation, regio-condensation and product hydrolysis, usually happen, resulting in the byproducts during N-glycan synthesis (Hamilton, 2004; Hancock et al., 2006; Faijes and Planas, 2007). To overcome this limitation, engineering of the GH active sites by mutagenesis could result in a glycosynthase, which provides improved practicability by blocking the hydrolysis ability while keeping the capacity of glycan transformation (Kittl and Withers, 2010). One successful case is ENGase, whose mutants have become a widely used tool to synthesize the homogeneous glycoproteins including therapeutic monoclonal antibodies (mAbs) in the past decade (Fairbanks, 2017). The typical procedure is accomplished by the combination of GH and glycosynthase; i.e., heterogeneous glycans are first removed by GHs, followed by the installation of structure-defined homogeneous N-glycans back to the glycoprotein by glycosynthase (Li et al., 2017a). In addition, some other GHs have also been engineered to generate the useful glycosynthases for the construction of N-glycans and glycopeptides (Perugino et al., 2004; Umekawa et al., 2008; Wang, 2008; Huang et al., 2009). For example, a highly efficient fucosynthase was generated by mutagenesis (N423H) of the 1,2-α-L-fucosidase from *Bifidobacterium bifidum*, which showed the ability to add fucose moieties to both N- and O-linked glycans on the asialofetuin (Sugiyama et al., 2017). A Golgi endo-α-mannosidase was mutated (E407D) to generate the glycosynthase, which was able to mediate the transglycosylation from Glc-α1,3-Man-α-fluoride to the acceptor Man_8_GlcNAc_2_-BODIPY, resulting in the high-mannose type dodecasaccharide Glc_1_Man_9_GlcNAc_2_ (Iwamoto et al., 2017).

### Sugar Donors in N-Glycosylation

For glycosylation reactions, except for GTs, another key issue is sugar donors. Most GTs use sugar nucleotides as donors, among which UDP-Glc, UDP-GlcNAc, UDP-Gal, GDP-Man, GDP-Fuc, and CMP-Sia are commonly found in the N-glycan biosynthesis pathway. Some GTs use dolichol phosphate sugar as their glycosylation donor, such as a few Alg ManTs in the ER lumen that use dolichol phosphate mannose (Man-P-Dol) as the activated sugar donor to elongate the glycan chain (Figure 3A; Maeda and Kinoshita, 2008). Although nucleotide sugar substrates are often commercially available, their expensive price has driven the exploration of large-scale preparation methods by many groups in the past few years (Tanaka et al., 2012). Some sugar nucleotides are highly unstable, such as CMP-Sia which is indeed prone to hydrolysis due to the additional electron-withdrawing effect of the carboxyl group (Gilormini et al., 2016). To solve this problem, multienzyme catalyzed one-pot reactions and *in situ* sugar nucleotide regeneration system have been developed for the *in vitro* experiments (Tsai et al., 2013; Yu and Chen, 2016; Yu et al., 2016, 2017; Liu et al., 2019). For example, as the widely used donor of SiaT, CMP-Neu5Ac could be one-pot synthesized from cytidine triphosphate (CTP) and N-Acetyl-D-mannosamine (ManNAc) or Neu5Ac analogs by using sialic acid aldolase and CMP-sialic acid synthetase (Yu et al., 2005b, 2006, 2009). In addition, as the sugar donor of some GTs (Man-P-dolichol for Alg3, Alg9, and Alg12; Glc-P-dolichol for Alg6, Alg8, and Alg10) (Figure 3A), polyprenol sugar phosphate is always difficult to prepare because of its insolubility and the difficulty in obtaining dolichol. Therefore, some works used lipid sugar phosphate with lipid tails of different dolichol analogs as the mimic donor. For example, the phytanyl phosphate mannose (Man-P-Phy) which was chemo-enzymatically synthesized as an alternative to Man-P-dolichol (Wilson et al., 1995), was applied to the extension of Man_5_GlcNAc_2_ to Man_9_GlcNAc_2_ by recombinant Alg3, Alg9, and Alg12 (Figure 3B; Li et al., 2019a).

**Figure 5:**
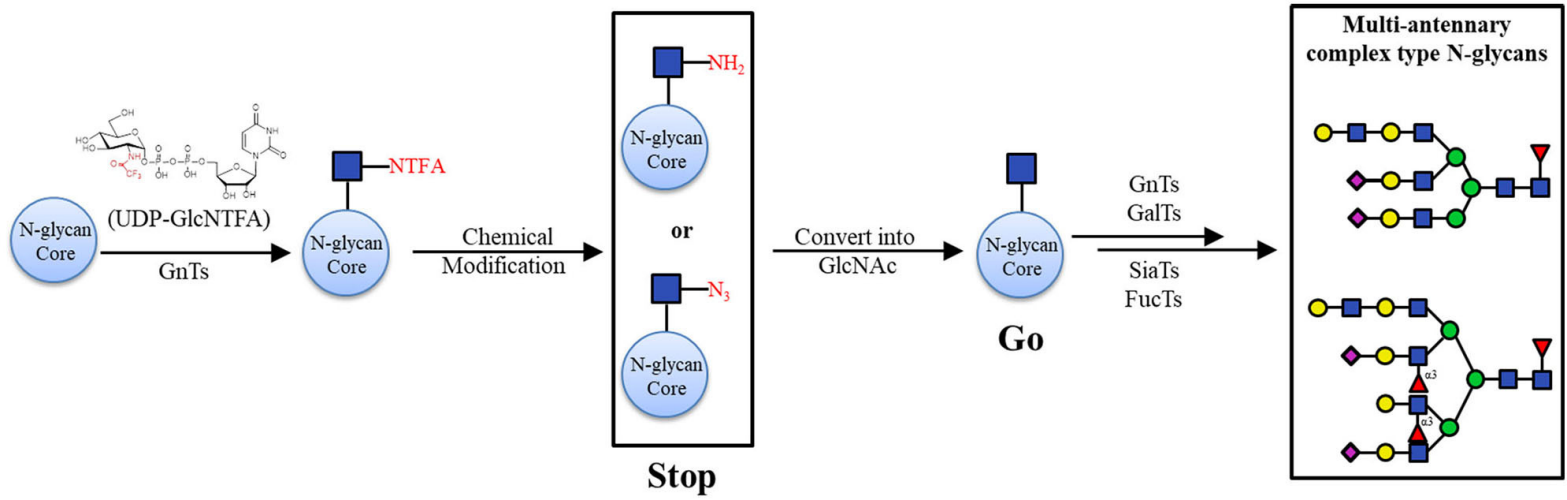
Chemo-enzymatic synthesis of asymmetric branched N-glycans with “stop and go” strategy. Transformation of GlcNTFA into GlcNH2 or GlcN3 (Stop); conversion of GlcNH_2_ or GlcN_3_ into GlcNAc (Go).

Furthermore, non-natural sugar donors have the capacity to be used in the synthesis of N-glycans. In 2004, a group synthesized some unnatural fucose nucleotides (UDP-Fuc, ADP-Fuc, CDP-Fuc) and evaluated their efficiency toward FucT-III (Khaled et al., 2004). The results showed that the kinetics of the conversion using these donor analogs were in the order: UDP-Fuc = ADP-Fuc > CDP-Fuc at a concentration of 20 mM, providing useful information on the enzyme specificities and structure-activity relationships. As an analog of the acetamido (-NHAc) group in N-acetylhexosamine-containing substrates, the trifluoroacetamido (-NHTFA) group was found to be an excellent substitute in enzymatic reactions (Sala et al., 1998). Boons and colleagues reported an off-the-rack biomimetic method for the synthesis of multi-antennary N-glycans with less than ten chemical and enzymatic steps. They use the non-natural sugar donor UDP-GlcNTFA, an analog of UDP-GlcNAc, to install GlcNTFA to the N-glycan core by recombinant GnTs (GnT IV and GnT V). The GlcNTFA moieties were chemically modified into GlcN_3_ or GlcNH_2_, which could not be further extended by GalTs, resulting in inhibition of galactosylation. At the appropriate step in the enzymatic elongation, these terminal GlcN_3_ and GlcNH_2_ species was converted into their natural GlcNAc counterparts to make the certain branching arms be elaborately processed into target constructs (Figure 5; Liu et al., 2019).

## Different Types of N-glycans Synthesized by Chemo-Enzymatic Methods

Chemo-enzymatic approaches to synthesize N-glycans combine the advantages of the flexibility of chemical methods and regio- and stereoselectivity of the enzymatic reactions. This highly efficient strategy starts from the chemical synthesis of key N-glycan modular structures or an N-glycan precursor from the natural source, followed by steps of enzymatic extension to achieve complicated N-glycan structures.

The pentasaccharide Man_3_GlcNAc_2_, which is shared in both DLOs and N-linked glycoproteins, is found in almost all eukaryotic cells. This structure is considered the key intermediate of N-glycans in both the *in vivo* biosynthesis pathway and *in vitro* synthesis strategies, which can be elongated and elaborated by various GTs, and is thus called a core pentasaccharide (boxed structure in Figure 3A). Moreover, Man_3_GlcNAc_2_ can be prepared from chemical synthesis or obtained from natural source digestion (Seeberger et al., 1996; Li et al., 2018b; Toonstra et al., 2018; Pistorio et al., 2019). Recently, the successful expression and purification of the ER ManTs Alg1 and Alg2 allow the reconstitution of the lipid-linked oligosaccharide (LLO) biosynthesis pathway up to the core pentasaccharide *in vitro* (Li et al., 2017b). Nevertheless, the availability of this chemo-enzymatic process is still limited due to the reaction of Alg2, which was only effective for LLOs with isoprenyl lipid chains longer than C20-C25 (Ramirez et al., 2017), making this step the major bottleneck in the synthesis of Man_3_GlcNAc_2_.

**Figure 6:**
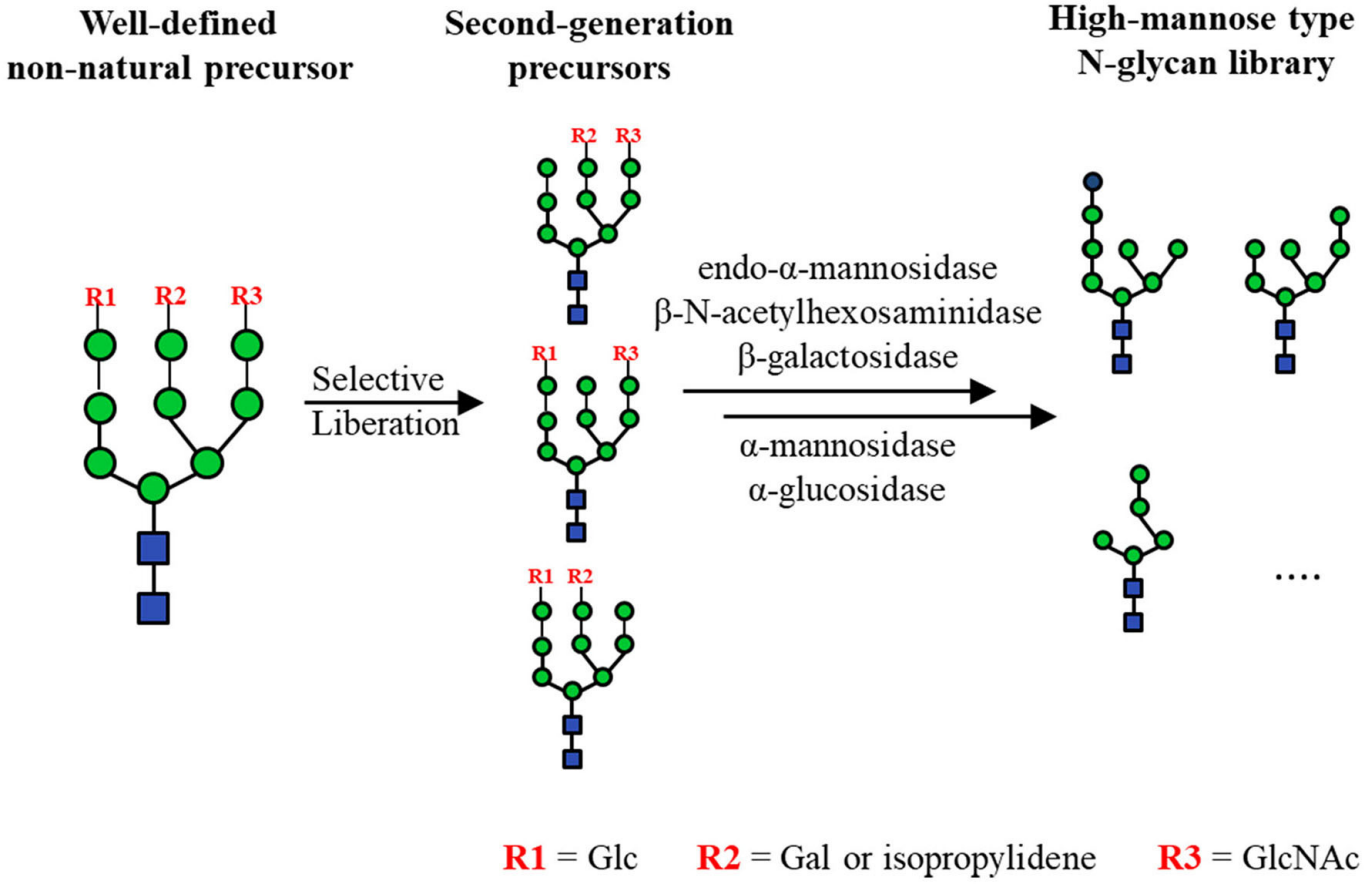
Chemo-enzymatic synthesis of high-mannose type N-glycan library by the orthogonal liberation of protection moieties in A, B and C arms from a non-natural tetradecasaccharide.

The core pentasaccharides can be extended to form high-mannose type N-glycans, which have been used to clarify the specificities of ER-related enzymes, such as calreticulin (Arai et al., 2005; Tatami et al., 2007), F-box protein Fbs1 (Hagihara et al., 2005), uridine 5'-diphospho-glucuronosyltransferase (Totani et al., 2005, 2009) and glucosidase-II (Totani et al., 2006). In addition to the various established chemical routes (Matsuo et al., 2003; Geng et al., 2004; Bailey and Bundle, 2014; Ramos-Soriano et al., 2017), several chemo-enzymatic synthesis methods have been developed to obtain high-mannose type N-glycans. Starting from LLO substrates with simplified lipid tails, a series of high-mannose type N-glycan precursors including Man_3_GlcNAc_2_ were produced through Alg-catalyzed elongation (Dsouza et al., 1992; Ramirez et al., 2017; Boilevin and Reymond, 2018; Li et al., 2018a; Rexer et al., 2018). More recently, the *in vitro* bottom-up chemo-enzymatic synthesis of full-length high-mannose type N-glycan Man_9_GlcNAc_2_ was accomplished by using recombinant Alg proteins expressed in *E. coli* and lipid-linked GlcNAc_2_ as the substrate (Li et al., 2019a; Figure 3B). Most Alg proteins have one or more transmembrane domains (TMDs), which lead to the difficulties in their expression and purification. To solve this problem, TMD truncated Alg1 and Alg11 were co-expressed with thioredoxin tagged Alg2, and the Alg3 and Alg9 were fused with Mistic-tag to generate Mistic-Alg3 and Mistic-Alg9. Without purification, the membrane fractions of *E.coli* were extracted and used for the construction of Man_9_GlcNAc_2_-PP-Phy. On the other hand, top-down chemo-enzymatic strategies were also practical in synthesizing high-mannose type N-glycans. Ito and coworkers chemically synthesized a well-designed high-mannose type N-glycan precursor whose terminal mannose moieties were selectively protected by a monosaccharide (i.e., Glc, GlcNAc and Gal) or a protecting group (i.e., isopropylidene), which were then trimmed by chemical deprotection or GH hydrolysis to give a library of high-mannose type N-glycans (Koizumi et al., 2013; Fujikawa et al., 2015; Figure 6). Two natural sources of N-glycans, i.e., Asn-linked Man_9_GlcNAc_2_ from soybean flour and SGP from chicken egg yolks, were also successfully treated by sequential enzymatic digestion to produce a library of N-glycans. These N-glycans could be conjugated to bovine serum albumin (BSA) to give neoglycoprotein microarrays for the comprehensive analysis of critical virus-neutralizing epitopes (Toonstra et al., 2018).

The structural diversity of N-glycans occurs during many cellular processes, such as embryogenesis, morphogenesis, cell cycle entry and oncogenesis (Freeze, 2006; Ohtsubo and Marth, 2006). Originating from the core pentasaccharides, the variation of N-glycan structures arises from the degree and patterns of LacNAcs on the branching arms, and further elaborates by the sialylation and fucosylation (Spik et al., 1985). Complex type N-glycans are usually substituted at each branching point, resulting in either symmetrical or asymmetrical architectures. In recent decades, scientists mainly focused on the synthesis of symmetrically branched complex type N-glycan structures by chemo-enzymatic synthetic strategies. For example, different human symmetrically N-glycans (e.g. GlcNAc_2_Man_3_GlcNAc_2_) were assembled from the bacteria-derived core pentasaccharide precursor with subsequent extension by different GnTs (Hamilton et al., 2017). In particular, isolated SGP from egg yolk, which can be trimmed by sialidases and β1, 4-galactosidase to give various complex type N-glycans with symmetrical branches have been widely used as the substrate (Huang et al., 2009; Li et al., 2016b; Wu et al., 2016).

**Figure 7:**
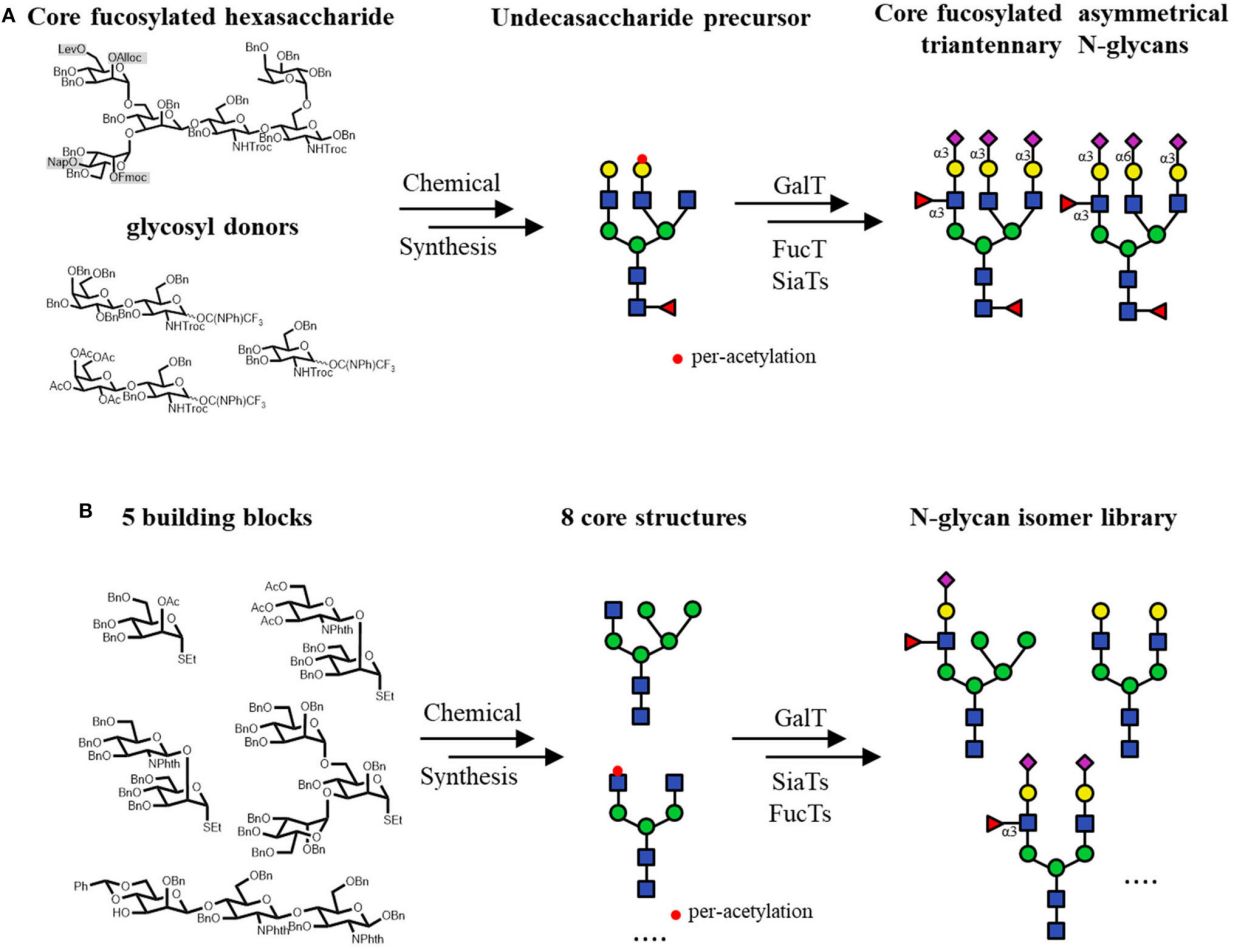
Core Synthesis/Enzymatic Extension (CSEE) strategy for N-glycan synthesis. **(A)** Chemo-enzymatic synthesis of core-fucosylated asymmetrical triantennary complex type N-glycans. **(B)** Construction of an N-glycan library by the enzymatic extension of 8 N-glycan core structures which are chemically synthesized from 5 building blocks.

Furthermore, several chemo-enzymatic strategies to access asymmetric N-glycans have also been established. A series of core-fucosylated asymmetric N-glycans were accomplished by the fucosylation with a *Caenorhabditis elegans* FUT8 expressed in *Pichia pastoris* (Serna et al., 2011). In this work, the chemical modular synthesized asymmetric N-glycan structures were core fucosylated with high efficiency in very short time, which sometimes required extensive work for the synthesis. In another study, libraries of asymmetrical multi-antennary glycans could be generated from the precursors by the selective extension. The attachment of GlcNAc, Gal, Sia and Fuc moieties was catalyzed by GnT, GalT, SiaT and FucT, respectively. To produce the precursor glycans, the core pentasaccharide Man_3_GlcNAc_2_ was modified at potential branching origin by orthogonal protecting groups, including levulinoyl (Lev), fluorenylmethyloxycarbonate (Fmoc), allyloxycarbonate (Alloc) and 2-naphthylmethyl (Nap) groups, which could be selectively deprotected and further chemically elongated with LacNAc and GlcNAc donors. This work greatly expands the availability of asymmetrical N-glycans, which are quite difficult to prepare from chemical methods or natural sources (Wang et al., 2013b). Similarly, to prepare core-fucosylated asymmetrical tri-antennary N-glycan isomers, an undecasaccharide was used as the precursor for enzymatic extension by GTs. The undecasaccharide precursor was synthesized from a core-fucosylated hexasaccharide with orthogonal protecting groups, which were sequentially chemical glycosylated with LacNAc and GlcNAc donors. The achieved fucosylated asymmetrical triantennary complex type N-glycans are suggested as the biomarkers of breast cancer (Figure 7A; Li et al., 2016a). In addition, a similar tetra-antennary asymmetric precursor, whose terminals were GlcNAc, LacNAc, non-native Gal-α-1,4-GlcNAc and Man-β-1,4-GlcNAc, was described as a substrate to prepare bi-, tri-, and tetra-antennary asymmetric N-glycans through enzymatic transformations (Gagarinov et al., 2017). One application of the above method was the assembly of some tri-antennary N-glycans of zona pellucida carrying sialyl Lewis X (SLe^x^) moieties at the C-2 and C-2' arms and a sialyl Lewis X-Lewis X (SLe^x^-Le^x^) residue at the C-6 antenna and another two analogs. The synthesized compounds were used to analyze the glycan-dependent interactions between human sperm and oocytes, indicating that the SLe^x^-Le^x^ residue is essential for the inhibiting effect, while the other SLe^x^ moieties showed much less effect (Chinoy et al., 2018).

When the abovementioned methodology was expanded to generate N-glycan libraries, it was named core synthesis/enzymatic extension (CSEE), which typically utilizes bacterial GTs and well-designed oligosaccharide core structures (Li et al., 2015; Wu et al., 2016; Calderon et al., 2017). An efficient CSEE strategy was developed to prepare N-glycans with or without (S)LeX moieties, which were rapidly purified by HPLC using an amide column to a minimum 98% purity at the milligram scale (Li et al., 2015). Eight N-glycan core structures with varying non-reducing-end GlcNAc residues were first chemically synthesized from 5 building blocks. Among them, 2 core structures had the peracetylated GlcNAc residue on either the α-1,6-Man or α-1,3-Man branch. After enzymatic extension on the unprotected branch, the acetyl protecting groups would be removed for the further elongation. At last, the 8 GlcNAc residues were elongated by a set of robust GTs to yield an N-glycan library of 73 structures in total (Figure 7B).

## Automatic Synthesis of N-glycans

Owing to the discovery of enzymes and the development of chemo-enzymatic synthesis techniques, it is supposed that most N-glycan structures can be prepared by GT and/or GH transformations. However, compared with the assembly of nucleotides and peptides, these approaches are still labor-intensive and time-consuming processes. The critical issue is probably that almost all peptides (Merrifield, 1965) and oligonucleotides (Caruthers, 1985) can be prepared by automated sequencing synthesis, even by non-specialists, but glycans cannot. In most cases, automated synthesis refers to the synthetic strategy based on a solid support, in which the enzymatic or chemical reactions occur on a solid-phase carrier such as beads, allowing simplified purification and washing steps.

Since Merrified and co-workers developed the solid-phase peptide synthesis (SPPS) method decades ago (Merrifield, 1965), much effort has been devoted to establish the convenient and efficient systems for the automated glycan synthesis (Schuerch, 1971; Plante et al., 2001; Ganesh et al., 2012; Nokami et al., 2013; Pistorio et al., 2016; Hahm et al., 2017; Panza et al., 2018; Wen et al., 2018). So far, automated synthesis of N-glycans mainly focused on the chemical strategies. For example, Seeberger and co-workers prepared the N-glycan core Man_3_GlcNAc_2_ using the automated solid-phase oligosaccharide synthesizer (Kröck et al., 2012). There are few examples of the automated chemo-enzymatic synthesis of the N-glyans. This could be due to the *in vitro* enzymatic synthesis of N-glycans commonly starts from a substrate containing the core pentasaccharide structure Man_3_GlcNAc_2_ (Figure 3A). To obtain the core pentasaccharide by enzymatic method, two ManTs Alg1 and Alg2, which have the specificity on the length of lipid tail of the LLO substrate, are required (Ramirez et al., 2017). Till now, the cost-effective large-scale enzymatic synthesis of Man_3_GlcNAc_2_ was not reported, thus it is difficult to get sufficient amount of this N-glycan core and immobilize it on the solid-phase carrier for automatic synthesis. An alternative method is to synthesize and immobilize the Man_3_GlcNAc_2_ core structure using chemical method for the automated chemo-enzymatic N-glycan synthesis, though there was no specific report in this field.

**Figure 8:**
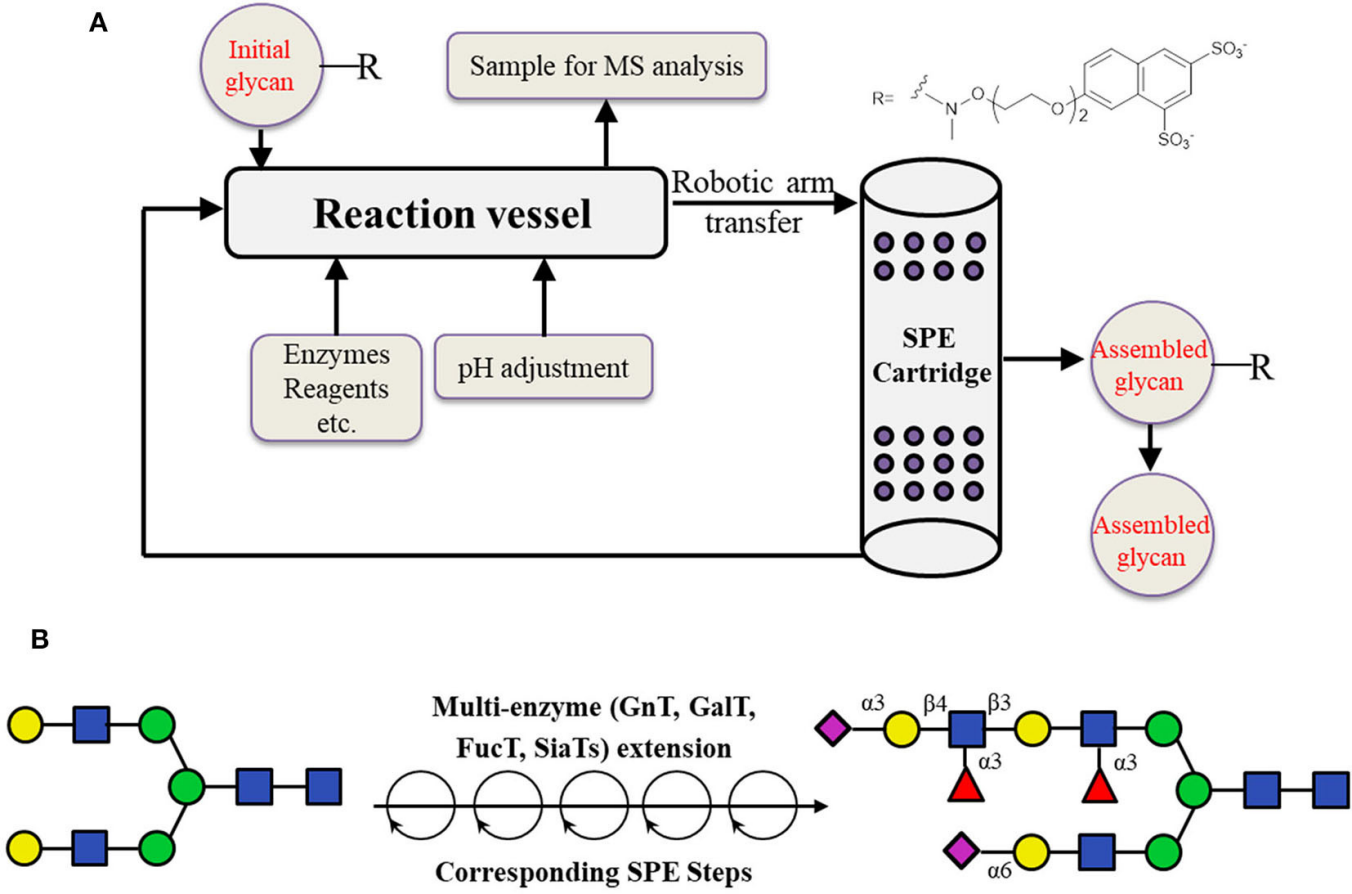
Enzyme-mediated assembly of N-glycans through the automated platform. **(A)** Schematic illustration of the automated oligosaccharide synthesizer. **(B)** Automated chemo-enzymatic synthesis of asymmetrical N-glycan from a naturally derived oligosaccharide precursor.

The other possible way is to prepare the desired core structure from natural precursors such as SGP from egg yolk by enzymatic digestion, and use it for the automatic chemo-enzymatic elongation after immobilization. Based on this method, an automated platform which can be used to automatically synthesize N-glycans was reported in 2019 (Li et al., 2019b; Figure 8). In this work, liquid-phase enzymatic reactions were first performed. The products were purified by being captured onto a resin selectively, followed by the release in appropriate conditions. The “catch and release” of glycan products were realized by introducing a sulfonate tag which could be easily installed and retrieved using a solid phase extraction (SPE) cartridge containing diethylaminoethyl (DEAE) resin. The products were eluted with aqueous ammonium bicarbonate, and the pH was adjusted with acetic acid to give an appropriate buffer for the next enzymatic reaction. In total, 15 reaction cycles were performed using this automated strategy, resulting in highly pure N-glycan structures (Li et al., 2019b).

In addition, various automatic devices for glycan synthesis have been developed, such as the HPLC-assisted automated synthesizer (Ganesh et al., 2012; Pistorio et al., 2016), the syringe pump-based electrochemical synthesizer (Nokami et al., 2013) and the automated glycan assembly (AGA) machine (Fair et al., 2015). These devices provide another option to chemo-enzymatically synthesize N-glycans and accomplish the purification of products.

## Applications of Chemo-Enzymatically Synthesized N-glycans

### Glycan Microarray

Immobilization of the glycans on the specific locations of a slide surface, the so-called glycan microarray, allows the high-throughput screening of carbohydrate-binding molecules (Oyelaran and Gildersleeve, 2009). As the discoveries on the significance and applicability of glycans in many biological processes have been continuously reported, glycan microarrays have attracted much attention. This technology not only has enabled comprehensive analyses of the interactions between glycan moieties and glycan binding proteins (GBPs) but could also be applicable for screening the binding properties of proteins, viruses, bacteria, yeast and mammalian cells (Geissner and Seeberger, 2016).

**Figure 9:**
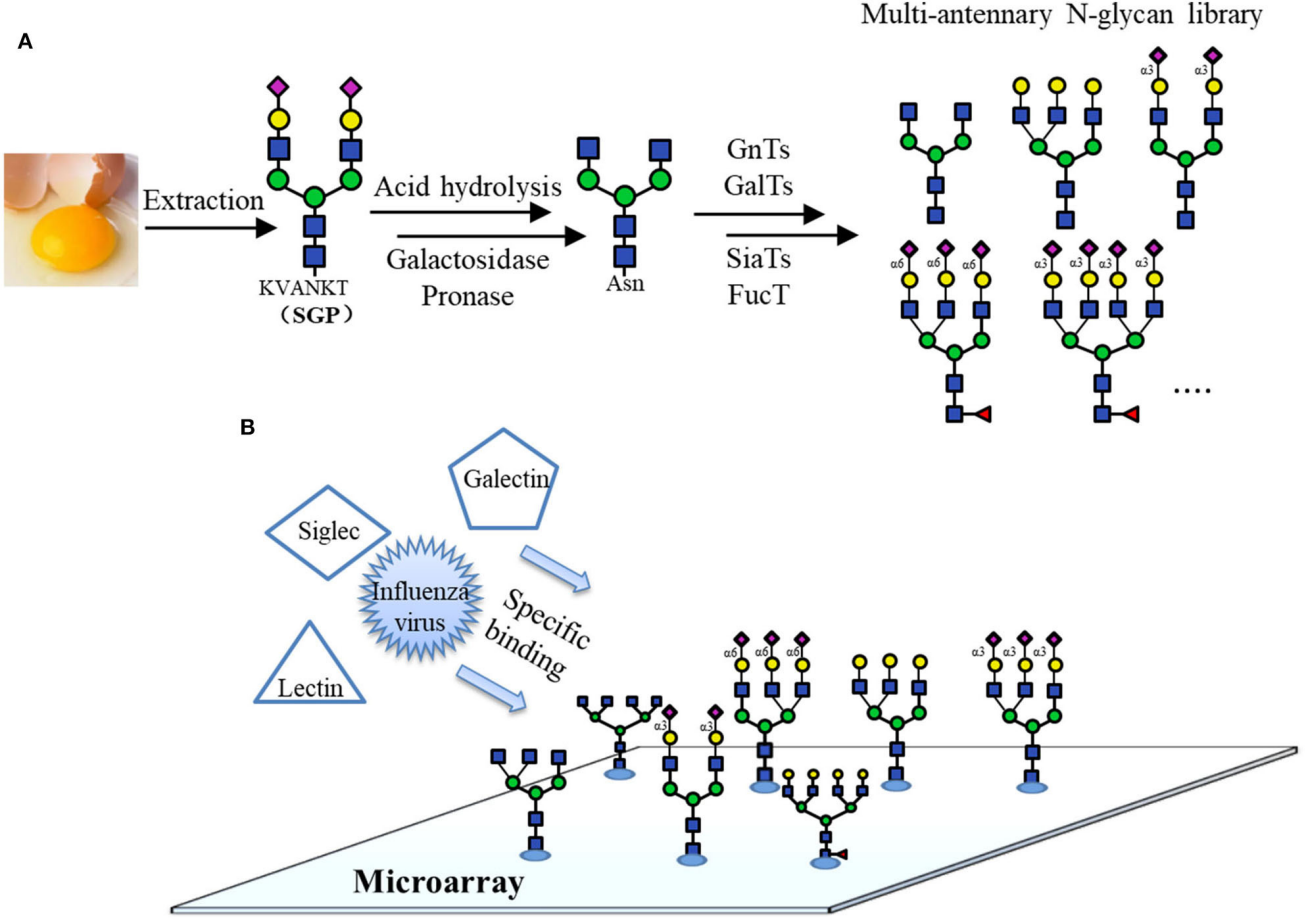
Microarray using chemo-enzymatically synthesized N-glycans. **(A)** Preparation of different naturally occurring complex type N-glycans by chemo-enzymatic methods from a common precursor obtained from egg yolk. **(B)** Binding specificity analyses of the lectins using N-glycan microarray.

Recently, several works based on chemo-enzymatically synthesized N-glycan microarrays have been reported. For example, it was used to rapidly screen and identify the optimal N-glycan structures recognized by broadly neutralizing antibodies (bNAbs), whose targets are the N-glycans on the HIV surface envelope glycoprotein GP120. Various N-glycans were prepared by the modular chemo-enzymatic synthesis and immobilized on an aluminum-oxide-coated glass slide (ACG). The detection of the HIV-1 bNAbs binding specificities for N-glycans using this microarray could accelerate the development of HIV-1 vaccines (Shivatare et al., 2016). In another work, a microarray of isomeric multi-antennary N-glycans varying in terminal Neu5Ac, Gal, GlcNAc and core Fuc synthesized by a chemo-enzymatic method was constructed (Gao et al., 2019; Figure 9A). Using this library of 33 N-glycans and 5 N-glycan conjugates, the specific recognition of plant lectins, human galectins, influenza viruses and Siglecs was investigated, providing new insights into the uses of lectins in glycan identification (Figure 9B).

### Homogeneous Glycopeptides and Glycoproteins

**Figure 10:**
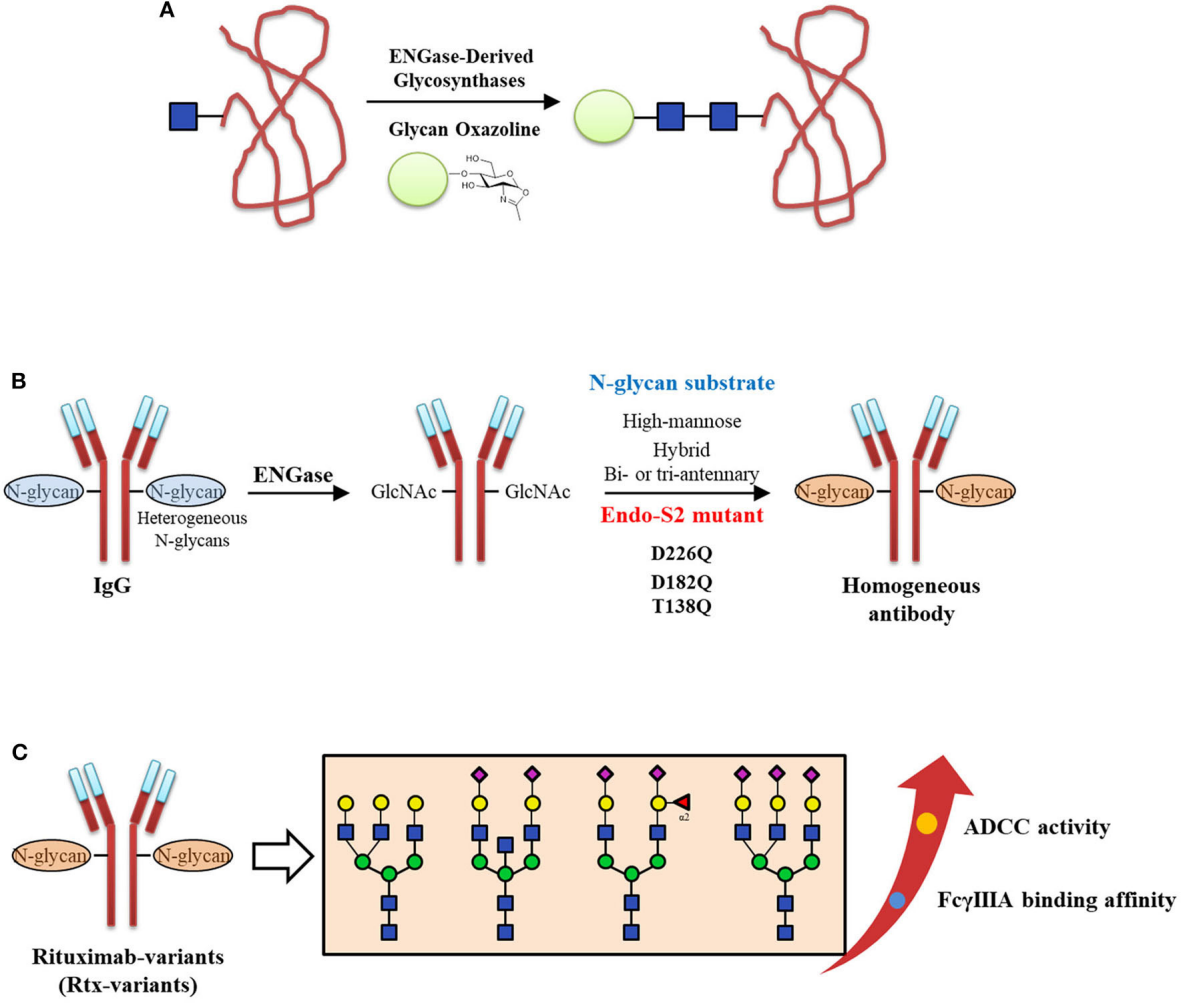
Chemo-enzymatic approach to prepare homogeneous antibodies. **(A)** The schematic diagram of synthesizing the homogeneous glycoproteins by ENGase-derived glycosynthases and glycan oxazolines. **(B)** Three identified Endo-S2 mutants (D226Q, D182Q, and T138Q) could be applied to transfer high-mannose, hybrid, bi- and tri-antennary complex type N-glycans to IgG. **(C)** The FcγIIIA binding affinity and ADCC activities of homogeneous Rtx-variants with different N-glycan structures.

N-Glycosylation of proteins with specific N-glycan structures is critical for their stability and biological functions (Jefferis, 2009). On the other hand, the high heterogeneity of N-glycans in glycoproteins makes it difficult to deep understand their structure and functional relationships and slows their use in therapy and diagnosis (Lowary, 2013). Thus, tremendous progress has been made to produce structurally defined homogeneous glycoproteins (such as antibodies), including both chemical synthesis methods (Pratt and Bertozzi, 2005; Kajihara et al., 2010; Unverzagt and Kajihara, 2013; Fernandez-Tejada et al., 2015; Seeberger and Overkleeft, 2015) and chemo-enzymatic methods (Bennett and Wong, 2007; Rich and Withers, 2009; Wang and Huang, 2009; Schmaltz et al., 2011; Wang and Amin, 2014; Danby and Withers, 2016; Fairbanks, 2017). In particular, endo-glycosidases such as ENGase have been well-developed and have become a useful tool to prepare homogeneous glycoproteins in the past decade (Fairbanks, 2017). In addition, the glycan oxazoline which can be utilized as the efficient donor of ENGase-catalyzed glycosylation reactions, were widely used in the chemo-enzymatic construction of various glycopeptides and glycoproteins (Zeng et al., 2006; Heidecke et al., 2009; Priyanka and Fairbanks, 2016; Yamaguchi et al., 2016). The synthetic strategy of the homogeneous glycoprotein by ENGase-derived glycosynthase and glycan oxazoline is shown in Figure 10A.

Traditionally, N-glycans used for the homogeneous glycoprotein preparation are chemically synthesized or isolated from natural sources, i.e., the bi-antennary complex type SGP from egg yolks and high-mannose type Man_9_GlcNAc_2_-Asn from soy bean flour. In 2009, two endo-glycosidase-based glycosynthases, EndoM-N175A and EndoA-N171A, were constructed to accomplish the assembly of homogeneous N-glycoproteins carrying natural N-glycans (Huang et al., 2009). Based on this work, the same group chemically synthesized glycan oxazolines bearing mannose-6-phosphate (M6P) moieties with different numbers and locations, which were transferred to the GlcNAc residue on the protein by ENGase, providing homogeneous glycoproteins with M6P-containing N-glycans (Yamaguchi et al., 2016).

Recently, chemo-enzymatic methods have become practicably in preparing the N-glycan structures used in homogeneous glycoprotein synthesis. For example, in a highly convergent chemo-enzymatic strategy, a large N-glycan oxazoline precursor was chemically synthesized and subsequently ligated to GlcNAc-RNase (bovine) catalyzed by EndoA-N171A, resulting in a glycoprotein with the selectively modified glycoform, i.e., GalGlcMan_9_GlcNAc_2_-RNase (Amin et al., 2011). After the terminal galactose was hydrolyzed by the β-galactosidase in excellent yield, the resulted monoglucosylated RNase GlcMan9GlcNAc2-RNase could serves as the specific ligand of calnexin and calreticulin. In 2018, Endo-S2 mutations were screened to give three mutants (D226Q, D182Q, and T138Q), which could transfer the high-mannose, hybrid-, and bi- or tri-antennary complex type N-glycans to prepare homogeneous Rituximab-variants (Rtx-variants) (Shivatare et al., 2018; Figure 10B). These Rtx-variants were used to examine the FcγIIIA binding affinity and further evaluate the antibody dependent cell mediated cytotoxicity (ADCC) activities (Figure 10C). This research investigated the glycosynthases possess various substrate specificities, significantly expanding the application of chemo-enzymatic approach to obtain the desired homogeneous antibodies.

The development of chemo-enzymatic methods for synthesizing various complex N-glycans and investigating glycosynthases have simplified the preparation of homogeneous glycoproteins. The availability of these homogeneous glycoproteins will be of great significance in investigating the effects and functions of N-glycans in glycoproteins.

### Potential Biomarkers

Commonly, alterations in protein glycosylation, including N-glycosylation, will affect the biological function, thus leading to the disorder of cells (Varki, 1993). Researches on glycomics have shown the biological significance of N-glycans in human disease, particularly in the study of tumor cells, and several diseases related to N-glycans that directly indicate biological changes have been identified. These N-glycan biomarkers might be used to estimate the developing risk, serve as the diagnosis tools, and monitor the progress and medication effects of a disease. Thus, chemo-enzymatic synthesis was applied to prepare these potential biomarkers due to its efficiency and variability *in vitro*.

Along with the development of the glycan analysis methodologies, such as the high-throughput technologies to analyze large quantities of samples, many aberrant N-glycans associated with diseases have been discovered. For instance, the fucosylation and sialylation levels are found significantly changed in the N-linked glycoproteins of cancer patients (Peracaula et al., 2008) and the high-mannose type N-glycan (Man_9_GlcNAc_2_) was found in serum of aggressive prostate cancer patients (Wang et al., 2013a). These aberrant N-glycans are considered as the biomarkers of corresponding diseases, and some of them have been achieved using chemo-enzymatic approaches.

For example, asymmetrical N-glycans containing sLe^X^, which are structures detected in serum glycoproteins of breast cancer patients, were prepared with a panel of glycosyl transferases. These synthesized N-glycans showed the potential in disease early diagnostic, and served as the specific therapeutic targets (Alley and Novotny, 2010; Li et al., 2016a). Similarly, a tetra-antennary N-glycan detected in the tissue of ductal invasive breast carcinoma patients was chemo-enzymatically synthesized from an asymmetric tetra-antennary intermediate precursor. This biomarker was considered as one of the most complex N-glycan structures ever discovered, thus difficult for the chemical synthesis. After it was prepared, this compound commonly used as the standard to analyze the quantity and structure of the glycans on the glycoproteins from biological samples using mass spectrometry, which would help to understand the metabolic or disease processes and be useful for the early disease diagnosis (Gagarinov et al., 2017).

## Summary and Outlook

N-glycans are a family of highly diverse oligosaccharide structures that are assembled and trimmed by GTs and GHs. The chemo-enzymatic approach to produce N-glycans, combining the advantages of chemical and enzymatic glycosylation methods, shows high specificity, mild reaction conditions and economic efficiency. Meanwhile, an increasing number of identified or commercially available N-glycosylation enzymes and large-scale preparation methods for monosaccharide donors provide strong support for the chemo-enzymatic synthesis of N-glycan structures. To date, all three types of structurally well-defined N-glycans (i.e., high-mannose, hybrid and complex types) have been generated by chemo-enzymatic strategies, starting from either chemically synthesized materials or isolated natural substrates. These N-glycans are applicable in the analysis of the interactions between GBPs and carbohydrates by microarray, preparation of homogeneous glycoproteins and assembly of potential N-glycan biomarkers. In addition, novel methodologies such as the automated solid-phase chemo-enzymatic synthesis of N-glycans are under investigation.

Since N-glycans play essential roles in biological pathways, exploratory studies to establish mature and convenient technologies for the chemo-enzymatic synthesis of N-glycan structures will be the frontier research. More likely, the development of automated synthesis devices and systems that are easily accessible will have a revolutionary impact on N-glycan preparation, thereby leading to an understanding of N-glycan functions in biological systems and illuminating N-glycan-related therapies.

## Author Contributions

QC and NW wrote the manuscript. YD contributed to the creation of most figures in this manuscript. Z-HC and M-HX contributed to the editing of this manuscript. NW and X-DG revised and edited the final manuscript. All authors contributed to reference collection, selection, and final proof.

## Conflict of Interest

The authors declare that the research was conducted in the absence of any commercial or financial relationships that could be construed as a potential conflict of interest.

## Abbreviations

ER: endoplasmic reticulum
GT: glycosyltransferase
GH: glycosidase
CAZy: The Carbohydrate-Active Enzymes database
DLO: dolichol-linked oligosaccharide
LacNAc: N-acetyllactosamine
SGP: sialoglycopeptide
ENGase: β-N-acetylglucosaminidase
ManNAc: N-Acetyl-D-mannosamine
LLO: lipid-linked oligosaccharide
TMD: transmembrane domain
CSEE: core synthesis/enzymatic extension
SPPS: solid-phase peptide synthesis
SPE: solid phase extraction
DEAE: diethylaminoethyl
AGA: automated glycan assembly
GBP: glycan binding protein
ACG: aluminum-oxide-coated glass slide
Rtx: Rituximab
ADCC: antibody dependent cell mediated cytotoxicity.
